# Supplementary material for: The simplest construction of single-site catalysts by the synergism of micropore trapping and nitrogen anchoring
Source: Nat Commun. 2019 Apr 10;10:1657. doi: 10.1038/s41467-019-09596-x (PMC6458126; doi:10.1038/s41467-019-09596-x)
Supplement: Supplementary file 1 — Supplementary Information [file 41467_2019_9596_MOESM1_ESM.pdf]

Supplementary Information

The simplest construction of single-site catalysts by the synergism of micropore trapping and nitrogen anchoring

Zhang et al.

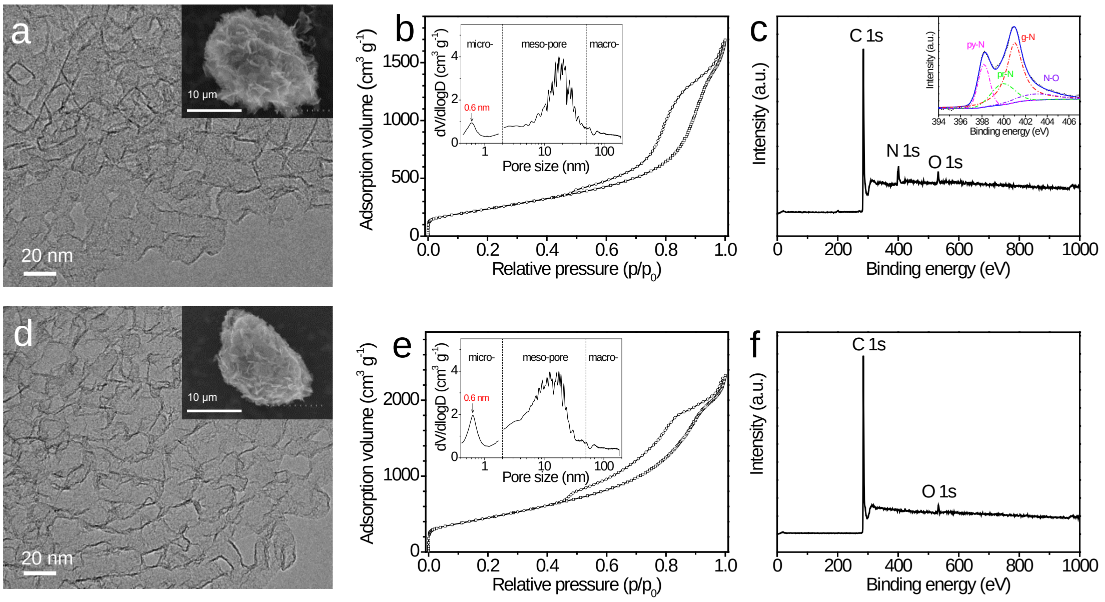

Supplementary Figure 1. Characterizations of the hNCNC and hCNC supports. (a-c) TEM image, N_2 adsorption-desorption isotherm, and XPS survey, respectively, for the hNCNC. (d-f) TEM image, N_2 adsorption-desorption isotherm, and XPS survey, respectively, for the hCNC. The insets in (a, d) are the corresponding SEM images, and in (b, e) the pore size distributions. The inset in (c) is the corresponding N1s XPS spectrum.

The hNCNC and hCNC supports possess the hierarchical morphology with coexisting micro-meso-macropores, and the corresponding specific surface area is 877 and 1650 m^2 g^{-1} respectively (a,b,d,e). The sizes of the micropores are ~0.6 nm (Insets in b and e). Nitrogen content is ~9.51 at.%, mainly existing as pyridinic N (py-N, 398.2 eV, 18.7%), pyrrolic N (pr-N, 399.9 eV, 18.2%), graphitic N (g-N, 401.0 eV, 53.9%), and pyridine-N-oxide group (N-O, 402.9 eV), respectively (c). The bulk conductivity of hNCNC and hCNC is about 84 and 463 S m^{-1}, respectively, measured by the four-wire method[1].

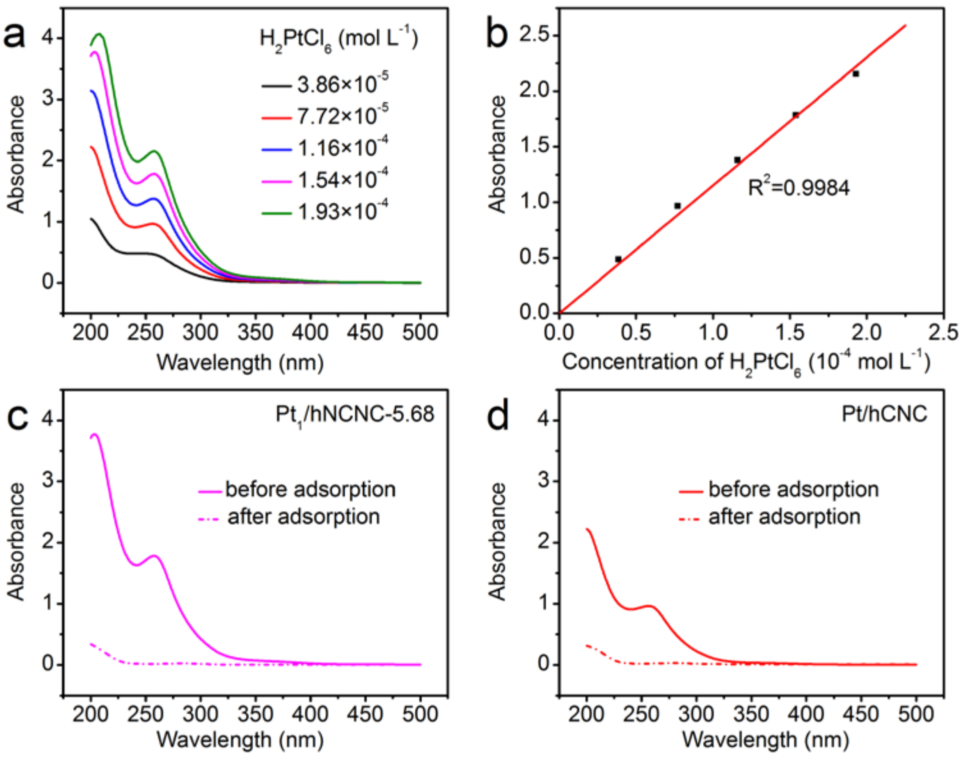

Supplementary Figure 2. UV-vis absorption measurements. (a,b) UV-vis spectra of H_2PtCl_6 aqueous solutions with different concentrations and the deduced working plot, respectively. (c,d) UV-vis spectra of solutions before and after adsorption for Pt_1/hNCNC-5.68 and Pt/hCNC, respectively.

After adsorption, little signal could be detected for the residual solution. This means the Pt-containing species is completely adsorbed by the hNCNC or hCNC supports. Hence, the Pt loading for Pt_1/hNCNC and Pt/hCNC in (c,d) can be obtained from the used amount of H_2PtCl_6 and support.

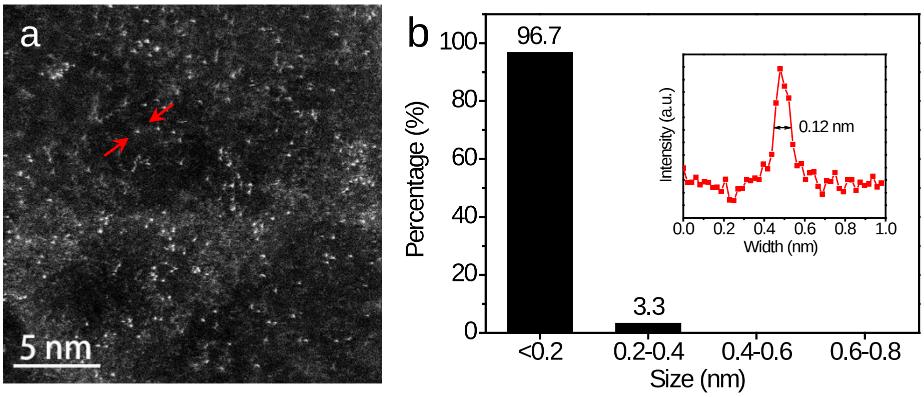

Supplementary Figure 3. HAADF-STEM characterization of Pt_1/hNCNC catalyst. (a) HAADF-STEM image. (b) The corresponding size distribution of Pt dots. The size distribution was determined by counting 1200 dots. The lineprofile inserted in (b) indicates that the size of a Pt atom is ~0.12 nm.

The size distribution in (b) reveals that the size of 96.7% Pt dots on hNCNC is less than 0.2 nm.

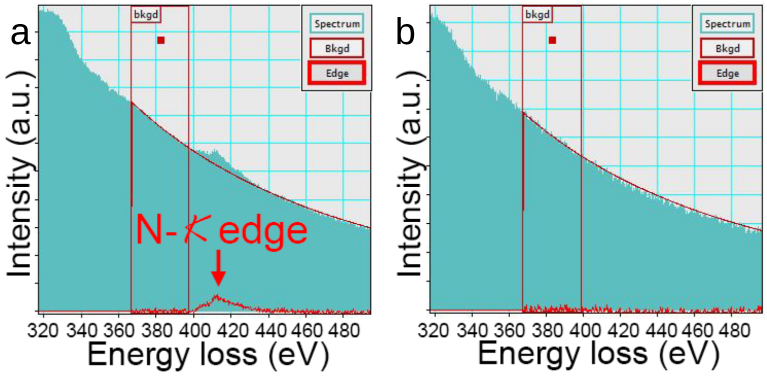

Supplementary Figure 4. Electron energy loss spectra. (a) Pt_1/hNCNC. (b) Pt/hCNC. The two spectra correspond to Fig. 1a,b in the main text, respectively.

As expected, the N signal is detected for Pt_1/hNCNC (a), while not detected for Pt/hCNC (b).

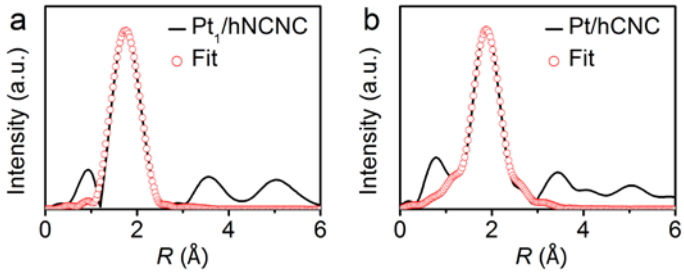

Supplementary Figure 5. k^3-weighted R-space Fourier transformed spectra from EXAFS and fitting curves. (a) Pt_1/hNCNC. (b) Pt/hCNC.

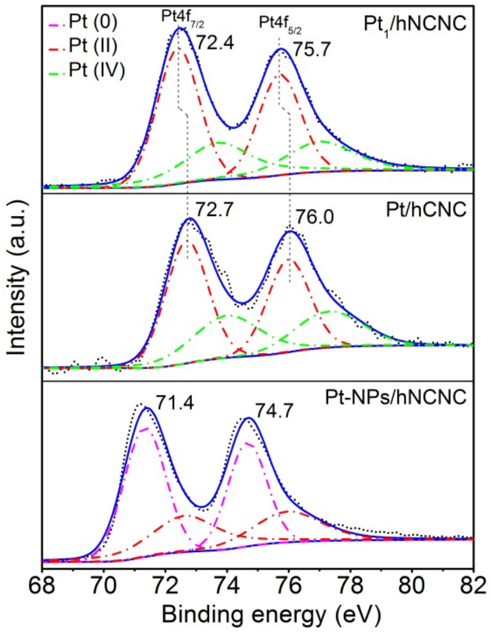

Supplementary Figure 6. XPS spectra of Pt_1/hNCNC, Pt/hCNC and Pt-NPs/hNCNC.

In comparison with the case of Pt-NPs/hNCNC, the Pt $4f_{7/2}$ and Pt $4f_{5/2}$ peaks for Pt_1/hNCNC and Pt/hCNC are positively shifted. This result indicates that Pt atoms in Pt_1/hNCNC and Pt/hCNC are partially oxidized.

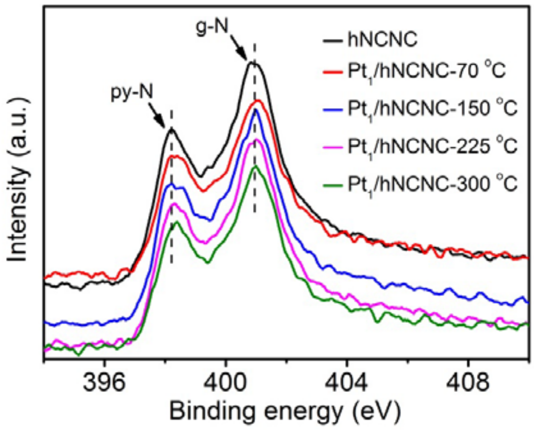

Supplementary Figure 7. N1s XPS spectra for Pt_1/hNCNC with different annealing temperatures.

The XPS signal of py-N in Pt_1/hNCNC shows slight widening to the higher binding energy side in comparison with that in pristine hNCNC support, suggesting the py-N anchoring effect for single Pt atoms. Actually, DFT calculation gives the much larger free energy for the adsorption of Pt atom on py-N than on g-N (Ref. 2).

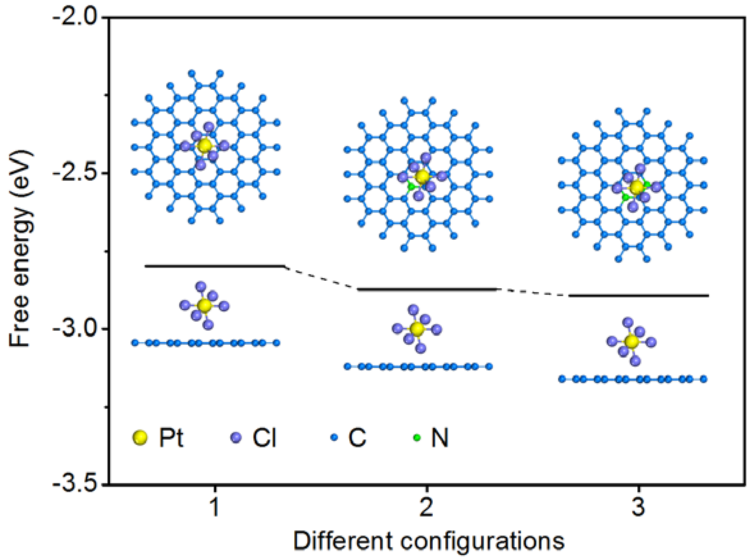

Supplementary Figure 8. The configurations and corresponding adsorption free energies of $[PtCl_6]^{2-}$ on different carbon supports. (1) graphene sheet, (2) graphene sheet decorated by one graphitic N atom, (3) graphene sheet decorated by two graphitic N atoms.

The graphitic N dopant has little contribution to the adsorption free energy, which is quite similar to the graphene sheet.

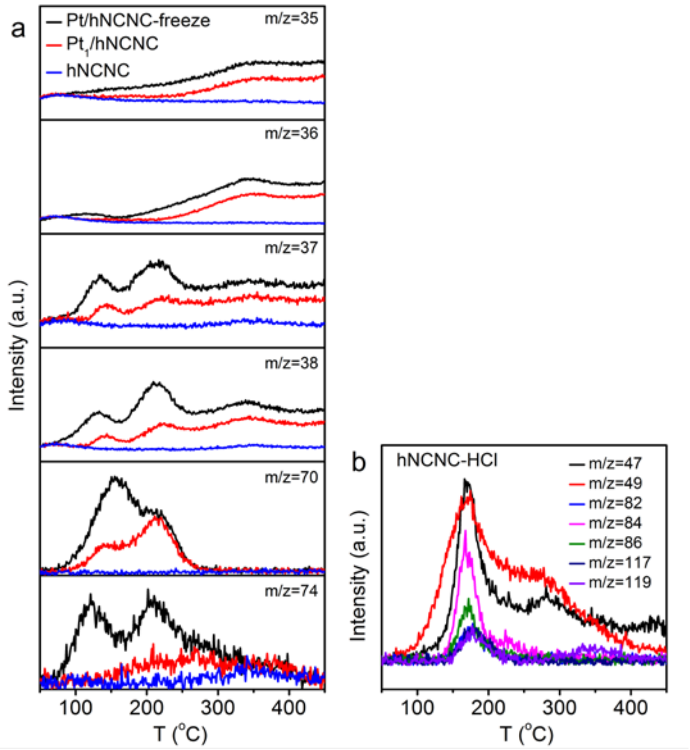

Supplementary Figure 9. Programmed-temperature mass spectrometry at a heating rate of 10 °C min^{-1} from 25 to 450 °C. (a) Pt_1/hNCNC, Pt/hNCNC-freeze and hNCNC. (b) hNCNC-HCl.
Note: m/z (mass-to-charge ratio) of 35 and 37 correspond to Cl; 36 and 38 to HCl; 70 and 74 to Cl_2; 47 and 49 to CCl; 82, 84 and 86 to CCl_2; 117 and 119 to CCl_3.

The preparation of Pt_1/hNCNC is described in the "Methods" in detail. Briefly, the solution of $H_2PtCl_6{\cdot}6H_2O$ was adsorbed on hNCNC by stirring at 70 °C for 10 h followed by a mild drying at 70 °C overnight. The control sample of Pt/hNCNC-freeze was prepared similarly but by stirring at 25 °C for 10 h followed by freeze-drying. The Cl-containing species in the Pt_1/hNCNC, Pt/hNCNC-freeze and hNCNC samples were examined and compared by mass spectrometry via a programmed temperature at the rate of 10 °C min^{-1} from 25 °C to 450 °C. Little signal of Cl-containing species exist for the hNCNC support. Hence, the Cl-containing species for Pt_1/hNCNC and Pt/hNCNC-freeze originate from $[PtCl_6]^{2-}$ anions. The Pt_1/hNCNC released the much less Cl-containing species than the Pt/hNCNC-freeze. This result implies the dechlorination occurs during the convenient synthesis of the Pt_1/hNCNC catalyst.

The Cl-related MS signals for Pt_1/hNCNC should be attributed to the Cl-containing species dissociated from the $[PtCl_6]^{2-}$ and partially adsorbed on hNCNC support during the synthesis at 70 °C. In other words, the signals don't come from the residual $[PtCl_6]^{2-}$. For supporting this point, hNCNC was immersed in the HCl solution (denoted as hNCNC-HCl), followed by filtrating, washing with distilled water and ethanol repeatedly, and drying at 70 °C. And then, the so-obtained hNCNC-HCl was detected by MS analysis upon heating. As shown in (b), the Cl species such as CCl, CCl_2 and CCl_3 were detected for hNCNC-HCl. This result indicates that the Cl-containing species can be adsorbed on the carbon support and then be desorbed/decomposed upon heating. Hence, in the synthesis of Pt_1/hNCNC, the Cl species dissociated from the $[PtCl_6]^{2-}$ could be adsorbed on the hNCNC, leading to the signals in the MS analysis. Actually, the dechlorination starts to occur at ca. 50 °C (below 70 °C) even at the programmed temperature condition (see the Pt/hNCNC-freeze sample in (a)). Our synthesis took a long time of 10 h under 70 °C, which should lead to the negligible residual $[PtCl_6]^{2-}$.

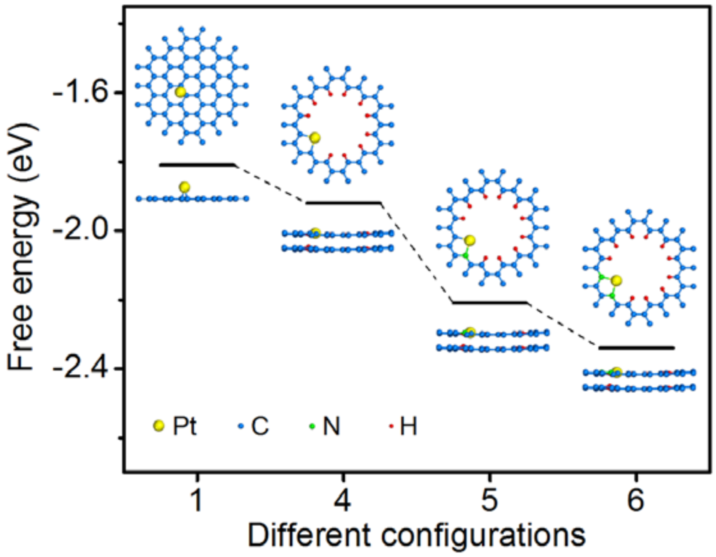

Supplementary Figure 10. Configurations of single Pt atom on different supports and corresponding adsorption free energies. 1. Graphene sheet; 4. Graphitic bi-layer with a micropore of 0.6 nm; 5. Graphitic bi-layer with the micropore decorated by one py-N atom; 6. Graphitic bi-layer with the micropore decorated by two py-N atoms.

In response to the models (4, 5, 6) in Fig. 2, the configurations of single Pt atom on graphitic bi-layer with a micropore (4, 5, 6) are presented. The configuration of single Pt atom on graphene sheet is presented as reference (Mode 1). The adsorption energy of the trapped single Pt atom on models (1, 4, 5, 6) are 1.81, 1.92, 2.21, 2.34 eV, respectively. Obviously, Pt single atoms trapped in hNCNC are more stable than the cases in hCNC or on graphene sheet.

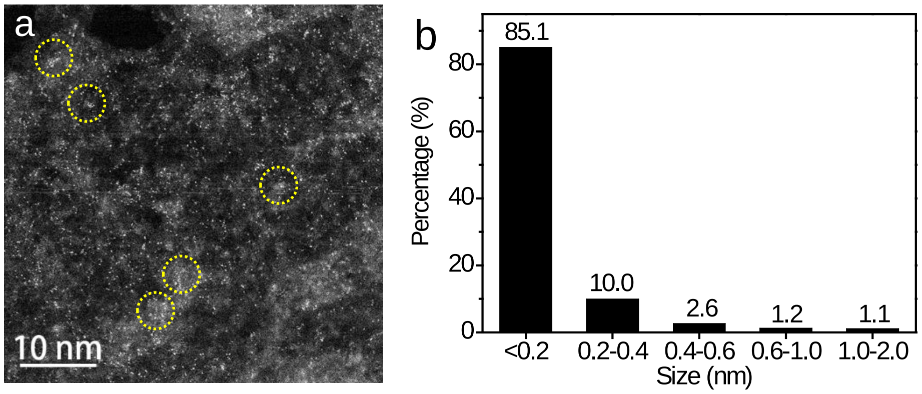

Supplementary Figure 11. HAADF-STEM characterization of Pt_1/hNCNC-5.68 catalyst. (a) HAADF-STEM image. (b) The corresponding size distribution of Pt dots. The size distribution was determined by counting 1200 dots.

The slight aggregation of Pt atoms in Pt_1/hNCNC-5.68 could be observed as circled, in comparison with the case of Pt_1/hNCNC-2.92 in Fig. 1a. In addition, the size of Pt dots under 0.2 nm decreases to 85.1%, and a little clusters in the size of 0.2-2.0 nm appeared.

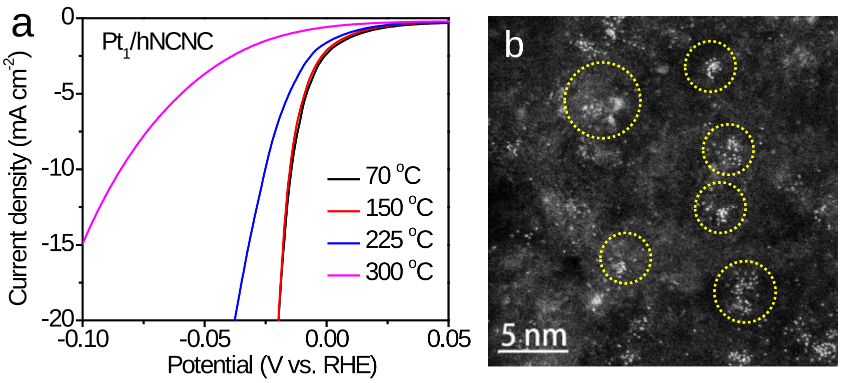

Supplementary Figure 12. Thermal stability of the Pt_1/hNCNC catalysts. (a) Polarization curves. (b) HAADF-STEM image after a heat treatment at 300 °C for 1 hour in Ar.

After additional heat treatment at 150 °C for 1 hour in Ar, the Pt_1/hNCNC catalyst remains the high HER activity (a). With a heat-treatment at 225 and 300 °C for 1 hour, the HER activity deteriorates remarkably with an overpotential increase from the initial 15 mV to 25 mV and 84 mV at 10 mA cm^{-2}, respectively, due to the aggregation of Pt atoms as circled in (b).

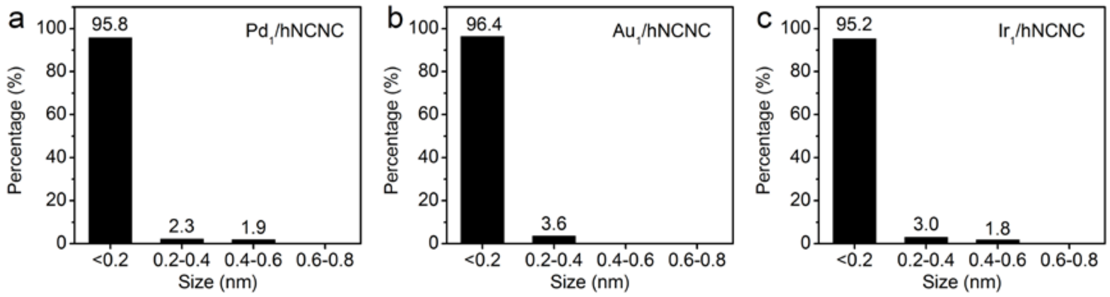

Supplementary Figure 13. The size distributions of different precious metals on hNCNC. (a) Pd. (b) Au. (c) Ir. The size distribution was determined by counting 1200 dots.

The size of over 95% dots (Pd, Au and Ir) on hNCNC is less than 0.2 nm, indicating the existence of high percentage of isolated single Pd, Au and Ir atoms.

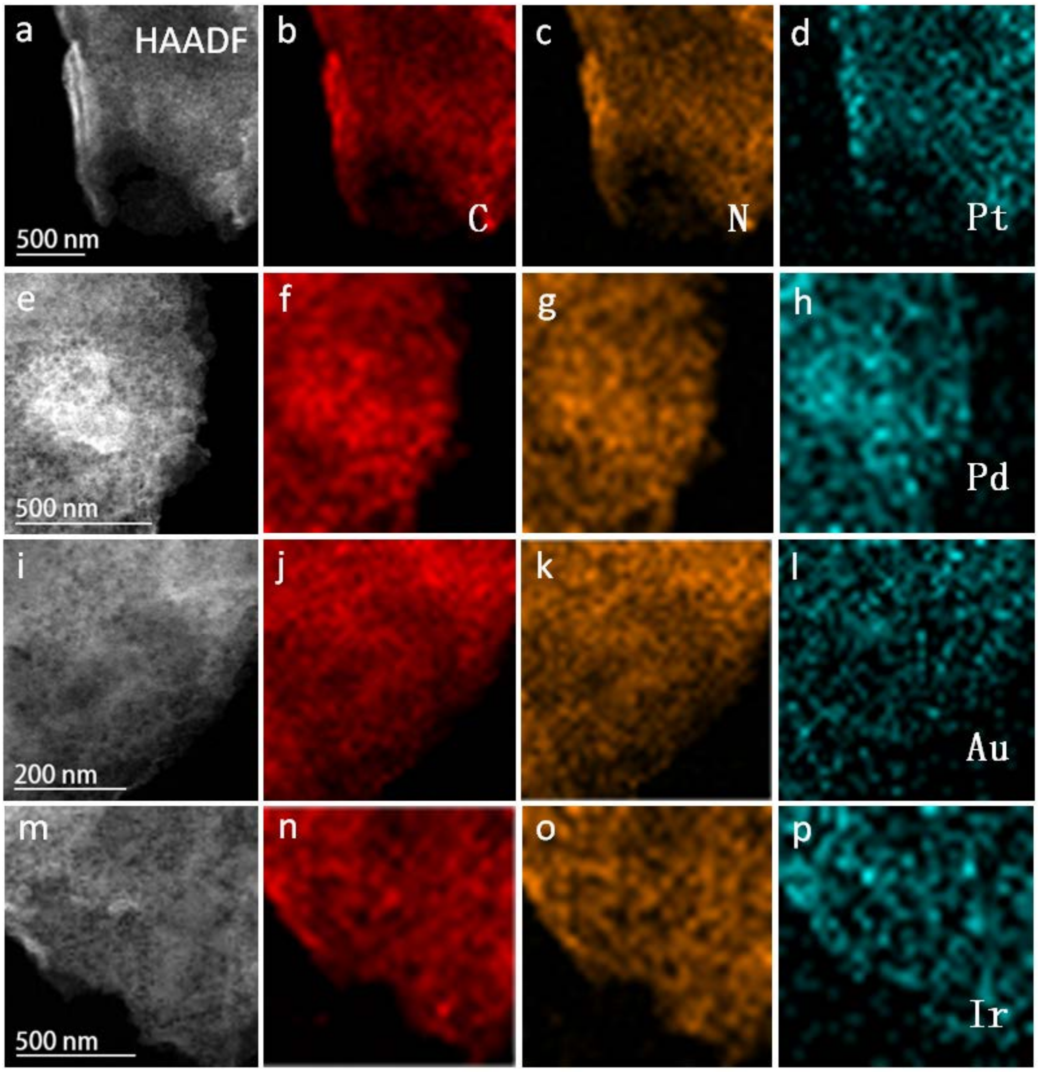

Supplementary Figure 14. The EDS elemental mapping of different single-site catalysts. (a-d) Pt_1/hNCNC. (e-h) Pd_1/hNCNC. (i-l) Au_1/hNCNC. (m-p) Ir_1/hNCNC. From left to right, the four columns are HAADF-STEM images and corresponding C, N and metal elemental mappings, respectively.

Supplementary Table 1. EXAFS parameters of Pt foil, Pt-NPs/hNCNC, Pt/hCNC and Pt_1/hNCNC.

Sample	Shell	N	R (Å)	σ^2 (10^{-3}Å^2)
Pt foil	Pt-Pt	12.0	2.76	4.72
Pt-NPs/hNCNC	Pt-Pt	8.0	2.75	6.34
Pt/hCNC	Pt-C_1/O	2.0	2.02	8.50
	Pt-C_2/O	2.0	2.49	1.59
	Pt-Pt	0.4	2.56	3.28
Pt_1/hNCNC	Pt-N	2.0	2.02	1.30
	Pt-C/O	2.0	2.48	12.0

Note:

N, coordination number with an error of 20%.

R, distance between absorber and backscatter atoms.

σ^2, Debye–Waller factor.

Supplementary Table 2. HER performance of Pt SSCs and Pt-based catalysts in acidic medium.

Sample	Overpotential (mV @ mA cm^{-2})	Tafel slope	Pt loading (mg cm^{-2})	Counter electrode	Ref.
Pt_1/hNCNC-2.92	15 @10 (29.5 @50)	24	0.00287	Graphite rod	This study
$Mo_2TiC_2T_x$-Pt_{SA}	30 @10	30	0.012	Graphite rod	3
Pt_1/mesoporous carbon	~25 @10 (65 @100)	26	0.01	Carbon rod	4
Pt-graphdiyne	66 @100	46.6	0.00465	Graphite rod	5
Pt-MoS_2	~130 @10	N.A.	0.01699	Carbon rod	6
Pt_1/NPC	25 @10	28	0.0038	Graphite rod	7
MoS_2@Pt	70 @10	36	0.01708	Graphite rod	8
$Pt_{13}Cu_{73}Ni_{14}$/CNF@CF	67 @5	38	N.A.	Graphite plate	9
Pt_1/MoO_{3-x}/C	23.3 @10	28.8	0.109	Graphite rod	10
ALD Pt on NGNs with 50 cycles	~45 @10	N.A.	0.00161	Pt wire	11
Pt nanoparticles over N,P co-doped carbon network	21.7 @20 (35 @100)	N.A.	0.00510	Pt foil	12
Pt-MoS_2	~53 @10	40	0.02701	Pt wire	13
Pt monolayer/Au NF/Ni foam	~95 @10	53	N.A.	Pt sheet	14
400-SWNT/Pt	~27 @10	38	~0.01942	Pt foil	15
WC@C@Pt	30 @10	26	0.0707	Pt flag	16
PtCoFe@CN	45 @10	32	0.01311	Pt wire	17
$Pt_{1.8}MoS_2$	80 @10	48	N.A.	Pt	18
Pt-CNSs/RGO	~75 @10	29	0.08033	Pt wire	19
Pt@N-doped hollow porous carbon	57 @10	27	0.00200	Pt wire	20
PtNiCu	25 @10	28	0.05421	Pt wire	21
$Pt_{66}Ni_{34}$ nanoflowers	43 @10	33	0.14699	Pt wire	22
Pt/MoS_2	86 @10	52	0.06600	Pt foil	23
$Pt_{2.6}Co_1$ nanoflowers	40 @10	42	0.15219	Pt wire	24

N.A.: not available.

Note: For the HER process, a graphite rod rather than a Pt wire should be used as the counter electrode, otherwise the transfer of Pt atoms from counter electrode to working electrode would result in the improved HER performance rather than the case of the catalysts themselves[25]. In spite of this, those HER performances based Pt counter electrode are also listed here for reference.

Supplementary References:

1 Lyu, Z. *et al.* Hierarchical carbon nanocages confining high-loading sulfur for high-rate lithium-sulfur batteries. *Nano Energy* **12**, 657-665 (2015).

2 Feng, H., Ma, J. & Hu, Z. Nitrogen-doped carbon nanotubes functionalized by transition metal atoms: a density functional study. *J. Mater. Chem.* **20**, 1702-1708 (2010).

3 Zhang, J. *et al.* Single platinum atoms immobilized on an MXene as an efficient catalyst for the hydrogen evolution reaction. *Nat. Catal.* **1**, 985-992 (2018).

4 Wei, H. *et al.* Iced photochemical reduction to synthesize atomically dispersed metals by suppressing nanocrystal growth. *Nat. Commun.* **8**, 1490 (2017).

5 Yin, X.-P. *et al.* Engineering the coordination environment of single-atom platinum anchored on graphdiyne for optimizing electrocatalytic hydrogen evolution. *Angew. Chem. Int. Ed.* **57**, 9382-9386 (2018).

6 Cheng, Y. *et al.* Rh-MoS_2 nanocomposite catalysts with Pt-like activity for hydrogen evolution reaction. *Adv. Funct. Mater.* **27**, 1700359 (2017).

7 Li, T., Liu, J., Song, Y. & Wang, F. Photochemical solid-phase synthesis of platinum single atoms on nitrogen-doped carbon with high loading as bifunctional catalysts for hydrogen evolution and oxygen reduction reactions. *ACS Catal.* **8**, 8450-8458 (2018).

8 Xu, X. Y. *et al.* Three electron channels toward two types of active sites in MoS_2@Pt nanosheets for hydrogen evolution. *J. Mater. Chem. A* **5**, 22654-22661 (2017).

9 Shen, Y., Lua, A. C., Xi, J. & Qiu, X. Ternary platinum–copper–nickel nanoparticles anchored to hierarchical carbon supports as free-standing hydrogen evolution electrodes. *ACS Appl. Mater. Interfaces* **8**, 3464-3472 (2016).

10 Liu, W. *et al.* Fabrication of a single-atom platinum catalyst for the hydrogen evolution reaction: a new protocol by utilization of H_xMoO_{3-x} with plasmon resonance. *ChemCatChem* **10**, 946-950 (2018).

11 Cheng, N. *et al.* Platinum single-atom and cluster catalysis of the hydrogen evolution reaction. *Nat. Commun.* **7**, 13638 (2016).

12 Wang, C. *et al.* 1.82 wt.% Pt/N, P co-doped carbon overwhelms 20 wt.% Pt/C as a high-efficiency electrocatalyst for hydrogen evolution reaction. *Nano Res.* **10**, 238-246 (2017).

13 Huang, X. *et al.* Solution-phase epitaxial growth of noble metal nanostructures on dispersible single-layer molybdenum disulfide nanosheets. *Nat. Commun.* **4**, 1444 (2013).

14 Li, M. *et al.* Pt monolayer coating on complex network substrate with high catalytic activity for the hydrogen evolution reaction. *Sci. Adv.* **1**, e1400268 (2015).

15 Tavakkoli, M. *et al.* Electrochemical activation of single-walled carbon nanotubes with pseudo-atomic-scale platinum for the hydrogen evolution reaction. *ACS Catal.* **7**, 3121-3130 (2017).

16 Liu, Z. *et al.* Thickness controllable and mass produced WC@C@Pt hybrid for efficient hydrogen production. *Energy Storage Mater.* **10**, 268-274 (2018).

17 Chen, J. *et al.* Enhanced activity for hydrogen evolution reaction over CoFe catalysts by alloying with small amount of Pt. *ACS Appl. Mater. Interfaces* **9**, 3596-3601 (2017).

18 Chia, X., Sutrisnoh, N. A. A. & Pumera, M. Tunable Pt–MoS_x hybrid catalysts for hydrogen evolution. *ACS Appl. Mater. Interfaces* **10**, 8702-8711 (2018).

19 Xu, G.-R., Hui, J.-J., Huang, T., Chen, Y. & Lee, J.-M. Platinum nanocuboids supported on

reduced graphene oxide as efficient electrocatalyst for the hydrogen evolution reaction. *J. Power Sources* **285**, 393-399 (2015).

20 Ying, J. *et al.* Nitrogen-doped hollow porous carbon polyhedrons embedded with highly dispersed Pt nanoparticles as a highly efficient and stable hydrogen evolution electrocatalyst. *Nano Energy* **40**, 88-94 (2017).

21 Cao, X. *et al.* Highly catalytic active PtNiCu nanochains for hydrogen evolution reaction. *Nano Energy* **9**, 301-308 (2014).

22 Huang, X.-Y. *et al.* Simple solvothermal synthesis of uniform $Pt_{66}Ni3_{4}$ nanoflowers as advanced electrocatalyst to significantly boost the catalytic activity and durability of hydrogen evolution reaction. *Electrochim. Acta* **271**, 397-405 (2018).

23 Ren, W., Zhang, H. & Cheng, C. Ultrafine Pt nanoparticles decorated MoS_2 nanosheets with significantly improved hydrogen evolution activity. *Electrochim. Acta* **241**, 316-322 (2017).

24 Jiang, L.-Y. *et al.* Facile solvothermal synthesis of monodisperse $Pt_{2.6}Co_1$ nanoflowers with enhanced electrocatalytic activity towards oxygen reduction and hydrogen evolution reactions. *Electrochim. Acta* **225**, 525-532 (2017).

25 Dong, G. *et al.* Insight into the electrochemical activation of carbon-based cathodes for hydrogen evolution reaction. *J. Mater. Chem. A* **3**, 13080-13086 (2015).
